# Down syndrome phenotype in a boy with a mosaic microduplication of chromosome 21q22

**DOI:** 10.1186/s13039-018-0410-4

**Published:** 2018-12-29

**Authors:** Franziska Schnabel, Mateja Smogavec, Rudolf Funke, Silke Pauli, Peter Burfeind, Iris Bartels

**Affiliations:** 1 Institute of Human Genetics, University Medical Center, Heinrich-Düker-Weg 12, 37073 Göttingen, Germany; 2 Department of Neuropediatrics, Sozialpädiatrisches Zentrum, Mönchebergstr. 41-43, 34125 Kassel, Germany

**Keywords:** Down syndrome, Down syndrome critical region, Microduplication, Mosaicism, *DYRK1A*

## Abstract

**Background:**

Down syndrome, typically caused by trisomy 21, may also be associated by duplications of the Down syndrome critical region (DSCR) on chromosome 21q22. However, patients with small duplications of DSCR without accompanying deletions have rarely been reported.

**Case presentation:**

Here we report a 5½-year-old boy with clinical features of Down syndrome including distinct craniofacial dysmorphism and sandal gaps as well as developmental delay. Conventional karyotype was normal, whereas interphase FISH analysis revealed three signals for DSCR in approximately 40% of lymphocytes and 80% of buccal mucosa cells. Array-CGH analysis confirmed a 2.56 Mb duplication of chromosome 21q22.13q22.2 encompassing *DYRK1A*.

**Conclusion:**

This presents one of the smallest duplications within DSCR leading to a Down syndrome phenotype. Since the dosage sensitive gene *DYRK1A* is the only duplicated candidate DSCR gene in our patient, this finding supports the hypothesis that *DYRK1A* contributes to dysmorphic and intellectual features of Down syndrome even in a mosaic state.

## Background

Down syndrome (DS), typically caused by complete trisomy 21, is associated with a wide range of clinical phenotypes including cognitive deficits, craniofacial dysmorphism, neurological, cardiovascular, immunological defects as well as ophthalmologic and hearing problems. DS is acknowledged as a gene dosage disease where overexpression of genes located on chromosome 21 distorts the balance of gene products. It has been known for decades that not the complete duplicated chromosomal region in trisomy 21 patients contributes equally to the DS phenotype. Particularly genotype-phenotype studies on partial trisomies performed by Sinet et al. [1] revealed that the genes which are essential in producing the main DS features cluster within a relatively small region in chromosomal bands 21q22.2q22.3. The minimum extent of this Down Syndrome Critical Region (DSCR), however, has been a matter of discussion. Until the year 2000, the technical options to establish a genotype-phenotype correlation were limited to targeted analyses and methods of restricted resolution, e.g. fluorescence in situ hybridization (FISH). Today, whole-genome array technologies allow to perform high-resolution breakpoint mapping and to rule out complex imbalances. DS patients with microduplications without concomitant imbalances are extremely rare. Here, we describe a 5½-year-old boy with a 21q22.13q22.2 microduplication in a mosaic state and distinctive clinical features of DS.

### Case presentation

**Fig. 1:**
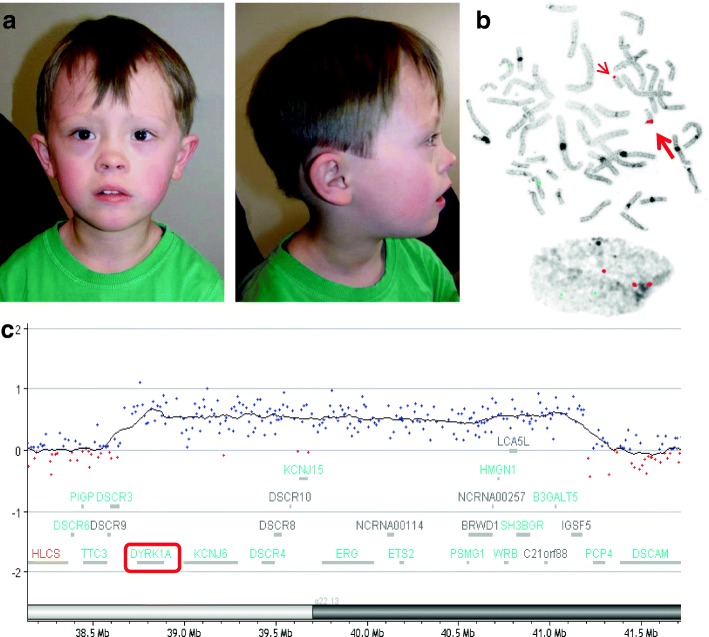
The facial appearance, FISH results and array CGH profile for the patient. **a** Frontal and lateral view of the patient at the age of 5½ years displays some distinctive clinical features of DS including posterior plagiocephaly, flat face, epicanthus, upslanted palpebral fissures and anteverted nares. **b** FISH result on metaphase spreads and interphase preparation using a probe for DSCR on chromosome 21 (orange signals) and RB-1 as a control probe on chromosome 13 (green). The fluorescence signal on one chromosome 21 is stronger as compared to the signal on the other chromosome 21 (arrows). Three orange signals for chromosome 21 are visible on interphase nuclei. **c** Array CGH result (Agilent, 180 K array). Zoom view of the 2.56 Mb mosaic microduplication and gene content at 21q22.13q22.2 encompassing *DYRK1A* (red box) as duplicated candidate DSCR gene

A 5½-year-old boy was referred to clinical genetic service for evaluation concerning DS. The patient is the first child of healthy non-consanguineous parents. His mother was 28 years old and his father was 31 years old when he was born. His younger sister showed no clinical abnormalities. The patient was born by vaginal delivery after a 9-week period of premature labor at 37 weeks of gestation with a birth weight of 3290 g (70th centile), length of 50 cm (50th centile) and occipitofrontal circumference of 32.5 cm (10th centile). Postnatally, the patient presented with muscular hypotonia, hyperopia and astigmatism. Throughout the first years a significant developmental delay was observed. Kiphard developmental test [2] was performed at the age of 36 months and 58 months in order to evaluate the stage of development at different levels. Optical perception was estimated as age-appropriate at both time points (39/36 and 58/58 months), whereas a delayed developmental age was observed in speech (34/36 and 45/58 months), auditory perception (35/36 and 42/58 months) and social contact (37/36 and 47/58 months). The most recent testing performed in the department of neuropediatrics at 6 years of age by Wechsler Preschool and Primary Scale of Intelligence (wppsi-III) showed a low-average overall IQ of 80. However, there were significant differences regarding specific abilities as expressive speech (VT-IQ = 88) reached just average score, whereas the ability to reason (HT-IQ = 81) and the processing speed (VG-IQ = 60) were in the low-average range. Motor development was quite normal, as he walked with support at 16 months and freely at 20 months. Echocardiography showed no abnormalities with a fractional shortening of 42%. At the age of 5½ years, his height was 113 cm (30th centile), his weight was 20.2 kg (50th centile) and his head circumference was 51 cm (25th centile). The patient presented with craniofacial dysmorphism including posterior plagiocephaly, flat face, epicanthus, upslanted palpebral fissures and anteverted nares (Fig. 1a). Furthermore he has a fused left upper lateral incisor and maxillary canine as well as sandal gaps.

## Methods

The karyotype of the patient was ascertained by standard cytogenetic analysis. Metaphase and interphase FISH analyses were performed using a DNA probe LSI 21 that binds to the DSCR region (21q22.13q22.2) and LSI13 RB1(13q14) as a control (Vysis, Abbott Molecular). The distribution of mosaicism was investigated on nuclei preparations from lymphocytes and non-cultured buccal cells by cohybridization of the two probes.

Genomic DNA was extracted from EDTA peripheral blood samples from the patient and his parents. The trio was investigated by microarray typing. Array comparative genomic hybridization (array-CGH) was performed on genomic DNA using the Human Genome 180 K CGH Microarray (Agilent Technologies, Waldbronn, Germany) according to the manufacturer’s recommendations. The results were scanned with the SureScan Dx Microarray scanner G5761A (Agilent Technologies, USA). Image analysis was carried out using the Feature Extraction Software V10.7 (Agilent Technologies, USA) and data analysis was performed using Cytogenomics V2.9.2.4 (Agilent Technologies, USA). Interpretation was based on Human Genome Assembly 37 (GRCh37/hg19).

## Results

Conventional chromosome analysis showed no numerical or structural aberration. Due to the persistent indication of DS, FISH was performed using a commercial DSCR-specific DNA probe. In approximately half of the metaphases a duplication was detected in the same cytogenetic band (karyotype: 46,XY.ish dup(21)(q22.13q22.2)(DSCR++)). Interphase FISH revealed a mosaic pattern with three signals for DSCR in approximately 40% of lymphocytes and in approximately 80% of buccal mucosa cells (Fig. 1c).

Array-CGH analysis detected a duplication at 21q22.13q22.2, spanning about 2.56 Mb (arr[GRCh37] 21q22.13q22.2(38668355_41187454)× 3 dn) (Fig. 1b), which was confirmed by qPCR. The duplication encompassed 15 OMIM genes (*DYRK1A*, *KCNJ6*, *DSCR4*, *DSCR8*, *KCNJ15*, *ERG*, *LINC00114*, *ETS2*, *PSMG1*, *BRWD1*, *HMGN1*, *WRB*, *SH3BGR*, *B3GALT5* and *IGSF5*). The duplication was not found in the parents of the patient.

## Discussion

In trisomy 21, not all loci are necessarily duplicated for the manifestation of DS. The concept of a DSCR on chromosome 21 is almost 30 years old [3]. Many studies were performed to define the minimum DSCR and identify those genes or contiguous genes whose overexpression provokes particular features of DS. It seems that duplications on chromosome 21 proximal from approximately 31 Mb [4] and distal from approximately 43 Mb [5] are not related to an abnormal phenotype.

Delabar et al. [6] narrowed down the critical region on chromosome 21q to a region between markers D21S17 and MX1, corresponding to approximately 35.89–41.72 Mb, whereas Lyle et al. [7] identified a minimum region of 37.94–38.64 Mb encompassing the genes *KCNJ6*, *DSCR4* and *KCNJ15* and a second one from the centromere to 26.96 Mb related to intellectual disability.

Most of the phenotype-genotype data for partial trisomy 21 were collected at pre-array days when additional imbalances due to unbalanced reciprocal translocations could not be ruled out [7, 8]. In this discussion, we focus on patients with pure microduplication of less than 5 Mb in size, which were investigated by array-CGH. All chromosomal coordinates were transformed to GRCh37(hg19). Microduplications with their respective sizes are summarized in Table 1. In addition to our patient, two other corresponding microduplications of chromosome 21q with a DS phenotype could be found in the literature [9, 10]. Furthermore, two microduplications of chromosome 21q without a DS phenotype have also been reported [11, 12]. Within the range of microduplication in 21q22 the physical size of the duplicated region itself does not seem to play a major role. While mother and daughter reported by Ronan et al. [9] show DS appearance and learning disabilities, mother and daughter reported by Su et al. [12] do not show this disease phenotype. Both families present with a 4.4 to 4.5 Mb duplication. Instead, the implementation of specific genes in the duplicated region is relevant for the phenotype.

**Table 1:** Summary of reported patients with pure microduplications 21q22.13q22.2 (< 5 Mb)

Publication	Size (Mb)	Segregation	DS phenotype	*APP*	*DSCR1*	*DYRK1A*	*DSCAM*
This Case	2.56	De novo	+	–	–	+	–
Ronan et al., 2007	4.3	Inherited from affected mother	+	–	–	+	–
Weisfeld-Adams et al., 2016	2.78	De novo	+	–	–	–	–
Eggermann et al., 2010	0.46	Inherited from healthy father	–	–	+	–	–
Su et al., 2006 (Case 1)	4.4	Inherited from healthy mother	–	–	–	–	–

Duplicated (+) and non-duplicated (−) classical DSCR genes (Delabar et al., [6]; Sinet et al., [1])

Candidate genes for the key clinical features of DS (classical DSCR) are *APP, DSCR1 (RCAN1), DYRK1A* and *DSCAM* [1, 6]. Our patient presented with a 2.56 Mb microduplication in the 21q22.13q22.2 region in a mosaic state which overlaps in part with the distal region of the 4.3 Mb microduplication in the three members of a family described by Ronan et al. [9]. Both microduplications encompass *DYRK1A* but none of the other classical DSCR genes (Fig. 1b and Table 1). The four patients share similar DS dysmorphic features and mild intellectual disability but no cardiac or other malformations.

*DYRK1A* (Dual Specificity Tyrosine Phosphorylation Regulated Kinase 1A) is a highly conserved proline-directed protein kinase with multiple domains, a high substrate diversity and known to be involved in neuronal processes [13, 14]. From mice data it was proposed that *DYRK1A* contributes together with *DSCR1* to the typical DS phenotype only in a synergistic way [15]. In both, the family studied by Ronan et al. [9] and our patient with DS phenotype, only *DYRK1A* is duplicated, but not *DSCR1*. Thus, the duplication of *DYRK1A* alone seems to be sufficient to impair normal intellectual development in those two families. *DYRK1A* belongs to the haploinsufficient genes on chromosome 21, meaning that loss-of-function mutations in *DYRK1A* can lead to a dominant form of intellectual disability [16]. On the assumption that haploinsufficient genes are sensitive to overexpression in partial trisomy, *DYRK1A* is a candidate gene for the intellectual phenotype in DS [17]. Overexpression of *DYRK1A* in transgenic mice induces dramatic deficits of serotonin levels in the brain [18] and has been shown to contribute to DS craniofacial dysmorphology [19].

All these findings on *DYRK1A* suggest that certain kinase inhibitors, such as polyphenol epigallocatechin-3-gallate, feature potential pharmacotherapy for cognitive impairment in DS. There are already therapeutic attempts and even clinical trials for the evaluation of *DYRK1A* kinase inhibitors [19–21]. Children with a microduplication involving *DYRK1A* such as described in our patient and the patients reported by Ronan et al. [9] could benefit from the development of new therapeutic options.

However, the pathogenesis of DS cannot be simply reduced to *DYRK1A*. Weisfeld-Adams et al. [10] reported a child with intellectual disability and distinct dysmorphic features of DS without heart defect. Array-CGH revealed a de novo 2.78 Mb microduplication on chromosome 21q22.11 involving none of the DSCR candidate genes. This duplication does not overlap with the duplicated region in our patient. This supports the hypothesis that more than the duplication of a single gene is causative for the DS phenotype. It seems that also other genes or other mechanisms contribute to the pathogenesis of DS. As further candidate genes, Weisfeld-Adams et al. [10] suggested *SOD1*, *SYNJ1* and/or *ITSN1,* which were included in the duplicated region in their patient.

In our patient the microduplication on chromosome 21q was present in a mosaic state. Mosaicism for trisomy 21 has been known since decades [22]. It has been even proposed that a low-grade mosaicism of trisomy 21 is present in the general population without any phenotypical resemblance of DS. Furthermore, the incidence of both, trisomy and microdeletions/−duplications in different tissues are underestimated and may contribute to different medical problems [23]. To our knowledge a mosaic microduplication with a DS phenotype has not been reported to date. The mosaic pattern of trisomic cells in our patient had a ratio of approximately 40% in lymphocytes and approximately 80% in buccal mucosa cells. A phenotype-genotype study showed that the trisomy level in mucosa cells is strongly correlated with phenotypic findings of ectodermal origin, whereas the trisomy level in lymphocytes is associated with heart malformations [24]. This might explain the intellectual impairment without heart phenotype in our patient.

## Conclusion

In summary, to the best of our knowledge this is the first report on a patient with a DS phenotype resulting from mosaicism for a microduplication of chromosome 21q. Since *DYRK1A* is the only duplicated candidate DSCR gene in our patient, this result reinforces the hypothesis that *DYRK1A* duplication contributes essentially to the intellectual phenotype even in a mosaic state. Current progress in the development of drugs such as *DYRK1A* kinase inhibitors may provide promising therapeutic options for such patients.

## Abbreviations

CGH: Comparative genome hybridization
DS: Down syndrome
DSCR: Down syndrome critical region
DYRK1A: Dual Specificity Tyrosine Phosphorylation Regulated Kinase 1A
FISH: Fluorescence in situ hybridization
